# A comparison of the neuroprotective efficacy of newly developed oximes (K117, K127) and currently available oxime (obidoxime) in tabun-poisoned rats

**DOI:** 10.1080/15376510802455362

**Published:** 2009-06-30

**Authors:** Jiri Kassa, Jana Zdarova Karasova, Kamil Musilek, Kamil Kuca, Young-Sik Jung

**Affiliations:** 1 Department of Toxicology, Faculty of Military Health Sciences, Trebesska, Hradec Kralove, Czech Republic; 2 Center of Advanced Studies, Faculty of Military Health Sciences, Trebesska, Hradec Kralove, Czech Republic; 3 Medicinal Science Division, Korea Research Institute of Chemical Technology, Yusong, Daejeon, Korea

**Keywords:** Atropine, Functional observational battery, Neurotoxicity, Oximes, Tabun

## Abstract

The potency of newly developed bispyridinium compounds (K117, K127) to reduce tabun-induced acute neurotoxic signs and symptoms was compared with currently available oxime (obidoxime) using functional observational battery. The neuroprotective effects of atropine alone and atropine combined with one of three bispyridinium oximes (K117, K127, obidoxime) on rats poisoned with tabun at a sublethal dose (180 μg/kg i.m.; 80% of LD_50_ value) were studied. Tabun-induced neurotoxicity was monitored using a functional observational battery and automatic measurement of motor activity at 24 h following tabun challenge. The results indicated that all tested oximes combined with atropine enabled tabun-poisoned rats to survive 24 h following tabun challenge while one tabun-poisoned rats died within 24 h after tabun poisoning when the rats were treated with atropine alone. Newly developed oxime K127 combined with atropine was the most effective in decreasing tabun-induced neurotoxicity in the case of sublethal poisonings among all oximes tested. Nevertheless, the differences of neuroprotective efficacy between K127 and obidoxime are not sufficient to replace obidoxime by K127 for the treatment of acute tabun poisonings.

## Introduction

Organophosphorus nerve agents are considered to be the most dangerous chemical warfare agents. These compounds pose potential neurotoxic threats to both military and civilian populations as evidenced by terrorist attacks in Japan (Ohtomi et al. 1996). Their acute toxic effects are based on the phosphonylation of acetylcholinesterase (AChE, EC 3.1.1.7), leading to the irreversible inhibition of its active site and subsequent overstimulation of postsynaptic cholinergic receptors due to the accumulation of the neurotransmitter acetylcholine in synapses of the central and peripheral nervous systems (Marrs 1993; Lotti 2000).

The medical countermeasures of nerve agent poisonings include the administration of the antidotes that are able to counteract the main toxic effects of nerve agents. The standard antidotal treatment of nerve agent poisoning usually includes an anticholinergic agent to block the overstimulation of cholinergic receptors and an oxime to reactivate nerve agent-inhibited AChE (Dawson 1994; Taylor 1996). The compounds with nucleophilic oximate anion were discovered and considered to be able to reactivate nerve agent-inhibited AChE by dephosphonylating the enzyme active site and restoring its activity. However, some nerve agents were found to be resistant to standard antidotal treatment. One of the most resistant nerve agents is tabun (O-ethyl-N,N-dimethyl phosphoramidocyanidate). Deleterious effects of tabun are extraordinarily difficult to antagonize because of the changes in hydrogen bonding and the conformational changes of AChE-tabun complex prior to an aging process in the AChE active site (Cabal and Bajgar 1999; Ekström et al. 2006).

Tabun can produce centrally-mediated seizure activity, that rapidly progresses to status epilepticus and contributes to profound brain damage (Marrs 1993; Taylor 1996). The exposure of experimental animals to tabun in convulsions-inducing doses may result in irreversible lesions in the central nervous system (CNS) that can be manifested as behavioral effects in survivors that have convulsed (Jokanovic 1993). Therefore, the ability of antidotes to block the acute neurotoxic effects of tabun and prevent development of irreversible lesions in the CNS is important for successful antidotal treatment. Generally, the oximes exert more potent effects in the peripheral compared to central system due to their poor penetration into the CNS. Nevertheless, there are published results demonstrating the penetration of oximes into CNS and subsequent reactivation of nerve agent-inhibited AChE in the brain (Cassel et al. 1997; Sakurada et al. 2003). Although the rate of the reactivation of nerve agent-inhibited AChE in the brain is lower compared to the peripheral system, the role of CNS is important for survival from nerve agent exposure (Marrs 1993; Kassa 2002).

**Figure 1:**
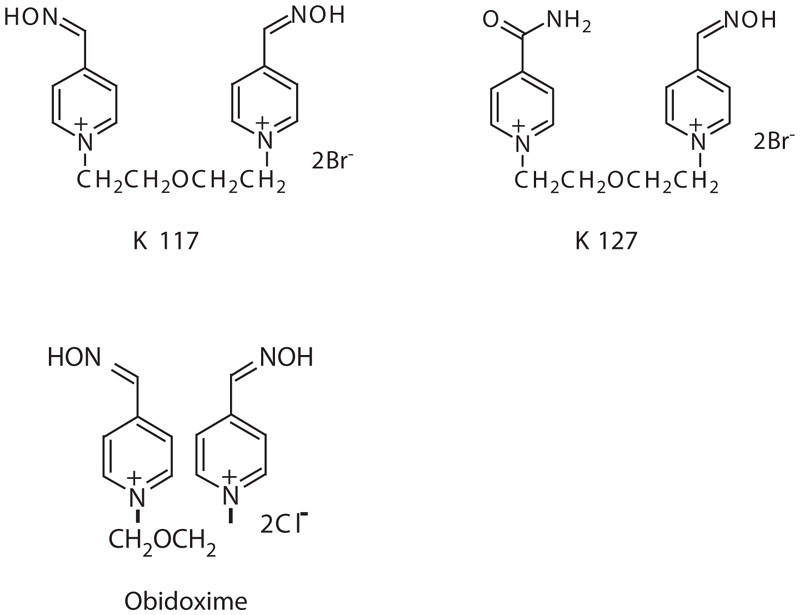
Chemical structures of oximes studied.

As the ability of currently used monopyridinium (e.g. pralidoxime) and bispyridinium oximes (e.g. obidoxime) to counteract the neurotoxic effects of tabun is generally poor (Kassa et al. 2005), the replacement of commonly used oximes with a more effective oxime has been a long-standing goal for the treatment of tabun poisoning (Dohnal et al. 2005). For this purpose, new bispyridinium oximes K117 [1,5-bis(4-hydroxyiminomethylpyridinium)-3-oxapentane dibromide] and K127 [1-(4-hydroxyiminomethylpyridinium)-5-(4-carbamoylpyridinium)–3-oxapentane dibromide] (Fig. 1) were synthesized (Kim et al. 2005; Musilek et al. 2006) to improve the efficacy of antidotal treatment in eliminating tabun-induced neurotoxicity.

The aim of this study was to compare the neuroprotective potency of newly developed oximes (K117, K127) with currently available oxime (obidoxime) in combination with an anticholinergic drug atropine in tabun-poisoned rats. The tabun-induced neurotoxic signs were determined using a functional observational battery, a non-invasive and relatively sensitive type of neurological examination for a wide range of neurobiological functions including measurements of sensory, motor, and autonomic nervous functions.

## Materials and methods

### Animals

Male albino Wistar rats weighing 200–230 g were purchased from Konarovice (Czech Republic). They were kept in an air-conditioned room (22 ± 2°C and 50 ± 10% relative humidity, with lights from 7.00 to 19.00 h) and allowed access to standard food and tap water ad libitum. The rats were divided into groups of eight animals. Handling of the experimental animals was performed in compliance with relevant laws and institutional guidelines and done under the supervision of the Ethics Committee of the Faculty of Military Health Sciences in Hradec Kralove (Czech Republic).

### Enzymes and chemicals

Tabun was obtained from Military Technical Institute in Brno (Czech Republic) and was 96% pure as assayed by acidimetric titration. All oximes studied of 98.5% purity were synthesized at the Department of Toxicology of the Faculty of Military Health Sciences in Hradec Kralove (Czech Republic). Their purities were analyzed using HPLC. All other drugs and chemicals of analytical grade were obtained commercially and used without further purification. All substances were administered intramuscularly (i.m.) at a volume of 1 mL/kg body weight (b.w.).

### In vivo experiments

Tabun was administered at a sublethal dose (180 μg/kg b.w. 80% LD_50_). One minute following tabun challenge, the rats were treated with atropine (21 mg/kg b.w.) alone or in combination with obidoxime, K117 or K127 in equitoxic doses corresponding to 5% of their LD_50_ values (Kassa et al. 2008). The neurotoxicity of tabun was monitored using the functional observational battery at 24 h following tabun poisoning. The evaluated markers of tabun-induced neurotoxicity in experimental animals were compared with the parameters obtained from control rats given saline instead of tabun and antidotes at the same volume.

The functional observational battery consists of 47 measurements of sensory, motor and autonomic nervous functions. Some of them are scored (Table 1), the others are measured in absolute units (Frantik and Hornychova 1995; Hornychova et al. 1995; Moser et al. 1997). The first evaluation was obtained when tabun-poisoned rats were in the home cage. The observer evaluated each animal's posture, palpebral closure, and involuntary motor movements. Then, each rat was removed from the home cage and briefly hand-held. The exploratory activity, piloerection, and other skin abnormalities were noted. Salivation and nose secretion were also registered and scored. Then, the rats were placed on a flat surface which served as an open field. A timer was started for 3 min during which the frequency of rearing responses was recorded. Rearing is the special kind of movement characterized by raising (both forelimbs are put up). This kind of movement is typical for exploratory behavior of rats. At the same time, gait characteristics were noted and ranked, and arousal, stereotypy, and bizarre behaviors and abnormal posture were evaluated. At the end of the third minute, the number of fecal boluses and urine pools on the adsorbent pad was registered. Reflex testing comprising recording each rat's response to the frontal approach of the blunt end of a pen, a touch of the pen to the posterior flank, and an auditory clic stimulus was also used. The response to a pinch on the tail and the ability of pupils to constrict in response to light were then assessed. These measures were followed by a test for the aerial righting reflex and by the measurements of forelimb and hindlimb grip strength, body weight, rectal temperature, and finally hindlimb landing foot splay. The whole battery of tests required approximately 6–8 min per rat. The observer of behavior did not know about the design of experiments. Motor activity data were collected shortly after finishing of the functional observational battery, using an apparatus for testing of a spontaneous motor activity of laboratory animals (constructed at the Faculty of Military Health Sciences, Hradec Kralove, Czech Republic). The animals were placed for a short period (10 min) in the measuring cage and their movements (total, horizontal, and vertical activity) were recorded.

**Table 1:** Functional observational battery (FOB).

	Scored values only									
	
Marker	−2	−1	0	1	2	3	4	5	6	7
Posture				sitting or standing	rearing	asleep	flattened	lying on side	crouched over	head bobbing
Catch difficulty				passive	normal	defense	flight	escape	aggression	
Ease of handling				very easy	easy	moderately difficult	difficult			
Muscular tonus	atonia	hypotonia	normal	hypertonia	rigidity	fasciculations				
Lacrimation			none	slight	severe	crusta	colored crusta			
Palpebral closure				open	slightly drooping	half-way drooping	completely shut	ptosis		
Endo-exophthalmus		endo	normal	exo						
Piloerection			no	yes						
Skin abnormalities			normal	pale	erythema	cyanosis	pigmented	cold	injury	
Salivation			none	sllight	severe					
Nose secretion			none	slight	severe	colored				
Clonic movements			normal	repetitive movements of mouth and jaws	nonrhythmic quivers	mild tremors	severe tremors	myoclonic jerks	clonic convulsions	
Tonic movements			normal	contraction of extensors	opisthotonus	emprosthotonus	explosive jumps	tonic convulsions		
Gait			normal	ataxia	overcompensation of hindlimbs movements	feet point outwards from body	forelimbs are extended	walks on tiptoes	hunched body	body is flattened against surface
Gait score				normal	slightly impaired	somewhat impaired	totally impaired			
Mobility score				normal	slightly impaired	somewhat impaired	totally impaired			
Activity				very low	sporadic	reduced	normal	enhanced	permanent	
Tension			none	partial (ears)	stupor					
Stereotypy			none	head weaving	body weaving	grooming	circling	others		
Bizarre behavior			none	head	body	self-mutilation	abnormal movements	others		
Approach response				no reaction	normal	slow reaction	energetic reaction	exaggerated reaction		
Touch response				no reaction	normal	slow reaction	energetic reaction	exaggerated reaction		
Click response				no reaction	normal	slow reaction	energetic reaction	exaggerated reaction		
Tail-pinch response				no reaction	normal	slow reaction	energetic reaction	exaggerated reaction		
Pupil size		miosis	normal	mydriasis						
Pupil response			no reaction	normal reaction						
Righting reflex				normal	slightly uncoordinated	lands on side	lands on back			

### Data analysis

Data collected with the functional observational battery and motor activity assessment include categorial, ordinal, and continuous values. Statistical analyses were performed on a PC with a special interactive program NTX (Frantik and Hornychova 1995). The categorial and ordinal values were formulated as contingency tables and judged consecutively by Chi-squared test of homogeneity, Concordance-Discordance test, and Kruskal-Wallis test, respectively. The continual data were assessed by successive statistical tests: CI for Delta, Barlett test for Equality of Variance, Williams test and Test for Distribution Functions (Roth et al. 1962). The differences were considered significant when *p* < 0.05.

## Results

Six of eight tabun-poisoned rats survived till the end of experiment (24 h following the intoxication). When tabun-poisoned rats were treated with atropine alone, seven of eight tabun poisoned rats survived within 24 h after tabun challenge. On the other hand, all tabun-poisoned rats treated with atropine in combination with one of the tested oximes survived till the end of experiment.

The results of the experiments related to the measurement of tabun-induced neurotoxicity at 24 h following tabun poisoning are divided into three parts (activity and neuromuscular measures, sensorimotor and excitability measures, and autonomic measures—Moser et al. 1997) and summarized in Tables 2–4. The observation of neurotoxic signs indicated that many functional disorders in poisoned rats lasted at least 24 h not only in tabun-poisoned rats but also in tabun-poisoned rats treated with atropine alone or in combination with K117 and obidoxime. Tabun produced passive behavior of rats during handling and retention, miosis, and a decrease in muscule tone at 24 h following tabun administration. The exploratory and rearing activity were significantly decreased and gait was severely impaired. In addition, no reaction during recording each rat's response to an auditory clic stimulus was observed. Non-treated tabun-poisoned rats and tabun-poisoned rats treated with atropine alone or atropine combined with K117 were not able to constrict their pupils in response to light due to tabun-induced miosis. A marked decrease in limb grip strength, food receiving, body temperature, and spontaneous horizontal as well as vertical motor activity were also observed at 24 h following tabun challenge (Tables 2–4). On the other hand, K127 in combination with atropine was able to prevent some tabun-induced signs of neurotoxicity observed at 24 h following tabun challenge with the exception of passive behavior of rats during handling and retention, miosis, gait impairment, a decrease in food receiving, body temperature, and spontaneous horizontal as well as vertical motor activity (Tables 2–4).

**Table 2:** The values of tabun-induced activity and neuromuscular neurotoxic markers measured at 24 h following tabun challenge by the functional observational battery (No 1–2, 4–14 scored values, No 3, 15–21 values in absolute units).

24 hours		Controls (*n* = 8)		Tabun-A + K117 (*n* = 8)		Tabun-A + K127 (*n* = 8)		Tabun-A + obidoxime (*n* = 8)		Tabun-A (*n* = 7)		Tabun (*n* = 6)	
						
No	Marker	*x/M*	± *s*	*x/M*	± *s*	*x/M*	± *s*	*x/M*	± *s*	*x/M*	± *s*	*x/M*	± *s*
1	posture	1.00		3.00*		3.00*		3.00*		3.00*		3.00*	
2	muscular tonus	0.00		−2.00*		0.00		0.00		−2.00*		−2.00*	
3	rearing	8.25	4.27	0.63*	1.77	5.00	5.71	1.63*	0.92	0.75*	1.75	2.25*	3.11
4	hyperkinesis	0.00		2.00*		0.00		5.00*		0.00		0.00	
5	tremors	0.00		2.00*		0.00		2.00*		0.00		0.00	
6	clonic movements	0.00		1.00*		0.00		1.00*		0.00		0.00	
7	tonic movements	0.00		3.00*		0.00		0.00		0.00		0.00	
8	gait	0.00		7.00*		5.00*		0.00		7.00*		1.00*	
9	ataxia	0.00		2.00*		0.00		0.00		0.00		2.00*	
10	gait score	0.00		2.00*		0.00		0.00		0.00		0.00	
11	mobility score	1.00		3.00*		1.00		1.00		1.00		1.00	
12	activity	4.00		1.00*		4.00		1.00*		2.00*		1.00*	
13	RRF	1.00		1.00		1.00		1.00		1.00		1.00	
14	RRV	1.00		1.00		1.00		1.00		1.00		1.00	
15	landing foot splay (mm)	94.00	12.47	78.63*	12.64	105.50	20.26	111.13*	15.97	89.31	40.57	59.88	39.66
16	forelimb grip strength (kg)	5.75	0.82	4.26*	1.06	5.39	1.11	5.98	0.90	5.13	2.05	4.95	1.01
17	hindlimb grip strength (kg)	1.14	0.22	0.49*	0.20	1.06	0.16	1.06	0.23	0.81*	0.31	0.67*	0.10
18	grip strength of all limbs (kg)	19.01	1.41	11.96*	2.92	19.55	3.79	20.23	2.40	12.93*	6.33	11.30*	2.71
19	vertical activity	121.00	112.29	16.75*	28.22	30.13*	38.04	10.50*	6.55	25.25*	29.49	30.13*	30.34
20	horizontal activity	12.63	17.82	1.88	4.55	2.63	3.11	0.13	0.35	3.25	7.23	3.50	7.21
21	total motor activity	133.63	127.29	18.63*	32.46	32.75	40.70	10.63*	6.67	28.50*	35.94	33.63	36.33

* *p* < 0.05 (comparison with the control values).

**Table 3:** The values of tabun-induced sensorimotor and excitability neurotoxic markers measured at 24 h following tabun challenge by the functional observational battery (scored values).

24 hours		Controls (*n* = 8)		Tabun-A + K117 (*n* = 8)		Tabun-A + K127 (*n* = 8)		Tabun-A + obidoxime (*n* = 8)		Tabun-A (n = 7)		Tabun (*n*= 6)	
						
No	Marker	*x/M*	± *s*	*x/M*	± *s*	*x/M*	± *s*	*x/M*	± *s*	*x/M*	± *s*	*x/M*	± *s*
1	catch difficulty	2.00		1.00*		1.00*		1.00*		1.00*		1.00*	
2	ease of handling	2.00		1.00*		1.00*		1.00*		1.00*		1.00*	
3	arousal (GSC)	1.00		4.00*		2.00*		1.00		4.00		2.00*	
4	tension	0.00		0.00		0.00		0.00		0.00		0.00	
5	vocalization	0.00		0.00		0.00		0.00		0.00		0.00	
6	stereotypy	0.00		0.00		0.00		0.00		0.00		0.00	
7	bizarre behavior	0.00		0.00		0.00		0.00		0.00		0.00	
8	approach response	2.00		2.00		2.00		2.00		2.00		1.00	
9	touch response	2.00		2.00		2.00		2.00		2.00		1.00*	
10	click response	2.00		3.00*		2.00		3.00*		2.00		3.00*	
11	tail-pinch response	2.00		2.00		2.00		2.00		2.00		2.00	

* *p* < 0.05 (comparison with the control values).

**Table 4:** The values of tabun-induced autonomic neurotoxic markers measured at 24 h following tabun challenge by the functional observational battery (Nos 1–7, 10–11, 15 scored values, Nos 8–9, 12–14 values in absolute units).

24 hours		Controls (*n* = 8)		Tabun-A + K117 (*n* = 8)		Tabun-A + K127 (*n* = 8)		Tabun-A + obidoxime (*n* = 8)		Tabun-A (*n* = 7)		Tabun (*n* = 6)	
						
No	Marker	*x/M*	± *s*	*x/M*	± *s*	*x/M*	± *s*	*x/M*	± *s*	*x/M*	± *s*	*x/M*	± *s*
1	lacrimation	0.00		0.00		0.00		0.00		0.00		4.00*	
2	palpebral closure	1.00		1.00		1.00		1.00		1.00		1.00	
3	endo/exophtalmus	0.00		0.00		0.00		0.00		0.00		0.00	
4	fur abnormalities	0.00		0.00		0.00		0.00		0.00		0.00	
5	skin abnormalities	0.00		0.00		0.00		0.00		0.00		0.00	
6	salivation	0.00		0.00		0.00		0.00		0.00		0.00	
7	nose secretion	0.00		3.00*		0.00		0.00		0.00		3.00*	
8	urination	1.25	1.58	2.25	4.46	0.75	1.49	2.88	3.60	1.00	1.29	1.17	2.40
9	defecation	0.00		0.00		0.00		0.00		0.00		0.00	
10	pupil size	0.00		−2.00*		−2.00*		−1.00*		−2.00*		−2.00*	
11	pupil response	1.00		0.50*		1.00		1.00		0.50*		0.00*	
12	food receiving (%)	100.00	0.00	20.50*	1.60	22.50*	2.67	34.00*	4.28	26.13*	10.84	37.50*	23.15
13	body weight (g)	243.38	16.80	282.88*	12.63	272.88	18.09	287.25*	14.00	223.86	19.55	227.50	12.29
14	body temperature (°C)	37.08	0.09	36.20*	0.56	36.47*	0.56	36.73*	0.35	36.54*	0.58	36.70*	0.45
15	respiration	0.00		0.00		0.00		0.00		0.00		0.00	

* *p* < 0.05 (comparison with the control values).

## Discussion

The potency of atropine alone to decrease tabun-induced acute neurotoxic signs is very low and corresponds to previously published results demonstrating that atropine alone is not able to prevent tabun-induced seizures and subsequent neurotoxic effects including brain damage following an exposure to tabun at sublethal and lethal doses (Kassa and Koupilova 2000; McDonough et al. 2000; Kassa and Kunesova 2006). Therefore, anticholinergic drugs such as atropine need to be combined with an AChE reactivator for more effective antidotal treatment of tabun poisonings. To combine atropine with an oxime, the efficacy of antidotal treatment of tabun poisonings is increased, although the central reactivating efficacy of oximes is lower compared to peripheral reactivating efficacy (Kassa 2002). Pralidoxime, a currently available oxime for the treatment of poisonings with highly toxic organophosphates (Dawson 1994), seems to be practically ineffective in preventing tabun-induced neurotoxicity (Kassa and Krejcova 2003). Another commonly used oxime (obidoxime) is able to partly eliminate tabun-induced acute neurotoxicity following i.m. administration of tabun at a lethal dose, nevertheless, its neuroprotective efficacy is not satisfactory (Kassa and Krejcova 2003; Kassa and Karasova 2007). The oxime HI-6 was demonstrated to be significantly less efficacious to block tabun-induced acute neurotoxicity than obidoxime (Kassa and Krejcova 2003; Kassa and Karasova 2007). The unsatisfactory efficacy of the above mentioned oximes to prevent tabun-induced acute neurotoxicity can be explained due to low potency of these oximes in reactivating tabun-inhibited AChE in vitro and in vivo (Puu et al. 1986; Jokanovic et al. 1996; Worek et al. 1998).

Our results demonstrate that the newly developed oxime K117 is completely ineffective to reduce tabun-induced acute neurotoxic signs and symptoms while the neuroprotective efficacy of another newly developed oxime K127 is markedly higher. The difference between neuroprotective efficacy of both newly developed oximes corresponds to the difference between their reactivating and therapeutic efficacy (Kassa et al. 2008). The low neuroprotective, reactivating, and therapeutic efficacy of K117 can be caused by low dosage due to its high acute toxicity (Kassa et al. 2008). The different toxicity of both newly developed oximes is probably caused by the differences in their chemical structure. The oxime K127 differs from K117 by the presence of carbamoyl group that diminishes its toxicity. On the other hand, the oxime K117 has two oxime groups that are responsible for its higher toxicity in comparison with K127.

The reason for a relatively high efficacy of K127 to reduce tabun-induced acute neurotoxic signs and symptoms is probably a chemical structure of its molecule because the potency of oximes to counteract the acute toxicity of nerve agents depends upon their chemical structure. The main structural features which influence their efficacy are the oxime functional group (its position and amount), the connecting linker for bisquaternary reactivators and other substituent(s) on the second heteroaromatic ring (Cabal et al. 2004; Kuca et al. 2006; Musilek et al. 2007). For tabun poisonings, at least one oxime in position four on the heteroaromatic ring is necessary for substantial reactivating, therapeutic, and neuroprotective potency, whilst an oxime in position two has a low or no capability to counteract acute toxicity of tabun (Kuca et al. 2006). Additionally, the optimal linker length suitable for tabun intoxication varies from three to four carbon–carbon bonds (Cabal et al. 2004). The neuroprotective efficacy of the oxime K127 is slightly better compared to obidoxime, although its therapeutic and reactivating efficacy does not prevail the effects of obidoxime (Kassa et al. 2008). Additionally, the difference of neuroprotective efficacy between K127 and obidoxime is not sufficient to replace currently used oximes by K127 for the treatment of acute tabun poisonings.
